# Identifying a structural brain network for social anxiety: connectome-based predictive modeling and network analyses in a transdiagnostic sample

**DOI:** 10.1038/s41398-026-04466-9

**Published:** 2026-09-28

**Authors:** Hannah Meinert, Marius Gruber, Emilia D. E. Brügge, Lisa Brüwer, Lisa Sindermann, Alexandra Winter, Theresa M. Slump, Fabian Breuer, Elisabeth Schrammen, Susanne Meinert, Janik Goltermann, Dominik Grotegerd, Jonathan Repple, Ronny Redlich, Linda M. Bonnekoh, Jochen Bauer, Udo Dannlowski, Joscha Böhnlein, Elisabeth J. Leehr

**Affiliations:** 1 Institute for Translational Psychiatry, University of Münster, Münster, Germany; 2 Goethe University Frankfurt, University Hospital, Department of Psychiatry, Psychosomatic Medicine and Psychotherapy, Frankfurt, Germany; 3 Goethe University Frankfurt, Cooperative Brain Imaging Center - CoBIC, Frankfurt, Germany; 4 Department of Psychiatry and Psychotherapy, University Hospital Bonn, Bonn, Germany; 5 Institute for Clinical Psychology and Translational Psychotherapy, University of Münster, Münster, Germany; 6 Institute for Translational Neuroscience, University of Münster, Münster, Germany; 7 Department of Psychiatry and Neuroscience, Campus Benjamin Franklin, Charité–Universitätsmedizin Berlin, Berlin, Germany; 8 German Center for Mental Health (DZPG), Site Berlin Potsdam, Germany; 9 Department of Biological and Clinical Psychology, Martin-Luther-University Halle-Wittenberg, Halle, Germany; 10 German Center for Mental Health (DZPG), Site Halle – Jena – Magdeburg, Germany; 11 LWL-University Hospital for Child and Adolescent Psychiatry, Ruhr-University Bochum, Hamm, Germany; 12 Department of Radiology, University of Münster, Münster, Germany; 13 Medical School and University Medical Center OWL, Protestant Hospital of the Bethel Foundation, Department of Psychiatry, Bielefeld University, Bielefeld, Germany; 14 Center for Intervention and Research on Adaptive and Maladaptive Brain Circuits Underlying Mental Health (C-I-R-C), Site Jena Magdeburg Halle, Germany; 15 Division of Clinical Psychology and Psychotherapy, University of Marburg, Marburg, Germany

**Keywords:** Predictive markers, Psychiatric disorders, Neuroscience

## Abstract

Trait social anxiety (SA) is continuous and can manifest across the entire spectrum of mental health, from subclinical levels in healthy controls to burdensome manifestations in major depressive disorder and finally full-blown disorders like social anxiety disorder (SAD). Although it has been proposed that SA is associated with deviations in the structural connectome, there is little research on the SA spectrum and its relation to abnormalities in brain structural networks. In this study, we aim to identify a structural brain network for SA across diagnostic groups using a transdiagnostic sample and connectome-based predictive modeling (CPM) to provide insight on neurostructural underpinnings of the SA spectrum. We collected magnetic resonance imaging (MRI) and clinical data of *N* = 160 participants across an SA spectrum. Diffusion-weighted and T1-weighted structural images were used to reconstruct each participant’s structural connectome. We applied CPM to identify a structural brain network that predicts SA across diagnostic groups. We further analyzed the structural connectome to investigate hubs and differences in global graph metrics between participants with and without SAD (noSAD *n* = 96, SAD *n* = 64). CPM revealed a brain network with 46 edges predicting SA across the SA spectrum with mean *r* = 0.141 (*CI* = [0.12, 0.2], *p* = 0.003). Hubs were located in the left pericalcarine, inferior-parietal, superior-parietal, right lateral-occipital cortex and pars orbitalis. This study provides first evidence for a shared neurobiological foundation of SA across diagnostic groups and points towards a transdiagnostic relevance of SA and the related brain network.

## Introduction

Trait social anxiety (SA) is a continuous phenomenon, with higher levels typically manifesting as social anxiety disorder (SAD). SAD is among the most prevalent anxiety disorders [1]. Without treatment, the course of SAD is usually chronic [2]. To better understand why some people are more socially inhibited than others and potentially disentangle trait SA-based manifestations, investigation of the neurobiological foundations of trait SA is warranted. This could improve the development and precise application of treatments, including prevention and early intervention by serving as a potential biomarker.

However, defining trait SA as a spectrum, especially in contrast to SAD, is complicated and has been subject of debate. SAD is characterized by excessive fear in and/ or avoidance of social situations, driven by the underlying concern to be embarrassed in front of and judged by others [3]. Though established as a diagnosis, distinction of SAD from personality traits, and subclinical manifestations is often debated [4, 5]. This might be because different levels of trait SA seem to occur over the full spectrum of mental health, including other mental disorders such as unipolar depression (MDD; 6, 7). Trait SA is characterized as a stable personality disposition involving heightened anxiety in social interactions and performance situations. It reflects a consistent tendency to experience discomfort or fear in social contexts, even if it does not meet the clinical threshold for a disorder [8]. Further, shared neural foundations of subclinical and clinically relevant anxiety have been reported previously.

While research on psychological processes underlying mental disorders as transdiagnostic factors has gained more and more attention [9–13], there is little to no research investigating the SA spectrum as a transdiagnostic trait [14–16]. Most studies investigating trait SA, its causes and consequences, use a categorical approach, comparing samples of HC to carefully selected participants with SAD (e.g. 17), as opposed to individuals across the spectrum of SA with and without mental disorders.

Although neuroimaging studies have implicated alterations in distributed neural circuits involved in emotional processing, salience detection, and cognitive control, with the most consistent functional findings involving the amygdala, insula, anterior cingulate cortex, and prefrontal regions, results remain heterogeneous. Furthermore, large-scale ENIGMA mega-analyses found no robust evidence for alterations in subcortical brain volumes in social anxiety disorder, suggesting that structural abnormalities are less consistent than functional alterations (for metaanalyses see 18–20), leaving the complex neural interplay related to SAD hardly understood. Network-theoretical approaches to brain-structural organization hold potential to address this issue due to their ability to assess not only characteristics of singular brain regions but rather their interplay by focusing on their interconnection. This approach is also proposed by Brühl et al. [18] in their meta-analysis due to the high complexity of the interaction of brain regions involved.

The structural network organization of the brain is referred to as the structural connectome and consists of all brain regions and their structural interrelations [21]. It can be investigated using diffusion tensor imaging (DTI) and T1-weighted magnet resonance imaging (MRI), which in combination reveal white matter fiber tracts connecting grey matter regions. It is hypothesized that healthy and adaptive behaviors and functioning are reflected in a correspondingly healthy structural connectome, whereas mental disorders might be the reflection of alterations in the structural connectome [22]. The degree of healthy and altered connectome organization can be represented by either local or global properties of the network itself, such as segregation and integration or centrality (for a detailed overview see 23). Applying this concept to trait SA and SAD could reveal shared and distinct neural endophenotypes for healthy and subclinical versus pathological social anxiety.

Still, the structural connectome has not yet been researched much in the context of anxiety disorders, and in SA specifically. Gruber et al. (in preparation) investigated the structural connectome of individuals with depression with and without comorbid anxiety and found an overall pattern of hyperconnectivity associated with anxiety. Zhang et al. [24] compared structural covariance networks in *N* = 53 HC and *N* = 49 patients with SAD using local and global graph metrics and found greater global efficiency, shorter characteristic path length, and stronger small-worldness in SAD, as well as differences in nodal degree in the left superior frontal gyrus, right superior parietal lobe, left amygdala, right paracentral gyrus, right lingual, and right pericalcarine cortex. Notably, they used support vector classification to predict group affiliation (SAD vs. HC) from grey matter covariance and achieved around 78% accuracy. Using DTI only, Liao et al. [25] found increased fiber density and mean fractional anisotropy in a seed region of interest (ROI) including the right medial prefrontal cortex (mPFC) and corpus callosum in SAD versus HC. Another study found significant increases in structural connectivity in a fronto-limbic network after 12 sessions of cognitive-behavioral group therapy in a sample with SAD compared to HC [26].

While these studies point towards an association of brain structural connectivity with measures of SA level or SAD duration not only compared to HC but also within SAD groups, only few studies have assessed connectivity in SA; to this day no studies have investigated the role of structural connectome in trait SA. Finally, connectivity studies investigating SA as a spectrum across several mental disorders do not yet exist. So far, neurobiological correlates of SAD have mostly been examined using fMRI [27–30]. This research gap has been pointed out in a systematic review, which included both studies with clinical as well as subclinical samples [31] and emphasized the potential multitude of (neural) SA endophenotypes, alluding to an SA spectrum. Still, due to the nature of the review, direct comparisons (e.g., between HC, subclinical SA and SAD) were not possible. However, there is a lack of studies explicitly focusing on this gap. Studying neural endophenotypes beyond fMRI and across the SA spectrum could provide valuable insights into its characteristics. It could also help to identify properties that are common across the entire SA spectrum, as opposed to those unique to clinically significant SAD, thus potentially serving as a more reliable biomarker for SAD.

This preregistered study (https://osf.io/fy3ac/) aims to investigate structural connectome signatures that are shared across the SA spectrum in a broad spectrum of symptom severity as well as categorial differences within these signatures between diagnostic groups. To this end, we applied the data-driven method of connectome-based predictive modelling (see 32, 33 for an introduction to the method) to a sample of HC and participants with mental disorders, including participants diagnosed with SAD and other disorders using the structural connectome. Using predictive methods in this context can help gain insight on the predictive value of a potential biomarker, and enforce generalizability and reproducibility of results.

Expanding on previous research, we hypothesized a significant association of overall SA level and structural connectivity across our transdiagnostic sample. Hence, we expected to identify a structural brain network, predicting the level of trait SA in our full sample (*H1*). Secondly, we aimed to characterize this SA-associated brain network by identifying its hubs (defined as a node that has many edges connecting it to other nodes in the network). Here, we expected hubs to be in line with previous research on neurostructural abnormalities associated with SA (*H2*). Thirdly, we investigated whether participants diagnosed with SAD (SAD subgroup) differ in graph-theoretical network characteristics from those without a diagnosis of SAD (noSAD subgroup) on a whole brain connectome level. We expected diverging global network metrics in the SAD group vs. the noSAD group (global efficiency, clustering coefficient, global connectivity strength and small-worldedness, *H3*).

## Method and materials

### Sample and procedure

The sample was part of the *Transdiagnostic Investigation of Emotional Processing in Depression and Anxiety* (TIP) cohort at of University of Münster (preregistered via OSF; https://osf.io/s6jqc/). Deviations from the pre-registered analysis plan are reported in a dedicated deviation protocol and in the relevant analyses sections of this paper. The study procedure was approved by the ethics committee of University of Münster (reference number: 2017-579-f-S). All participants signed written informed consent and were compensated for their participation.

General inclusion criteria were an age between 18 and 65, proficiency in German and right-handedness. Exclusion criteria were neurological abnormalities and related diseases, contraindications for MRI, and non-Caucasian descent. Participants in the SAD subgroup had to fulfill DSM-V criteria for SAD. Of the *N* = 64 participants in the SAD group, *N* = 49 also fulfilled the criteria for acute or (partly) remitted MDD. Participants in the noSAD group did not fulfill DSM-V criteria of SAD and had varying levels of trait SA. Within the noSAD group (N = 96), which included participants without current SAD but not necessarily without a lifetime psychiatric history, *N* = 49 participants reported no lifetime history of any mental disorder. *N* = 47 had a lifetime diagnosis of MDD (acute/ remitted). Other and comorbid mental disorders were excluded in both subsets, except for mild cannabis or alcohol abuse.

Participants were interviewed with the German version of the Structural Clinical Interview for DSM-IV-TR (SCID, 34), filled in a battery of questionnaires, including the Liebowitz Social Anxiety Scale (LSAS, Stangier & Heidenreich, 35), and completed neuropsychological testing, blood sampling and MRI. TMS and EJL verified post-hoc that all participants would have also fulfilled DSM-5 criteria for their main diagnosis (lifetime MDD or SAD respectively). All diagnostic data was obtained by trained personnel. A total of *N* = 160 participants were included in our analyses. See Table 1 for further sample characteristics and Supplementary Fig. 1 for a full study flow chart. Sample overlap with other projects is reported in Supplementary Material 2.

**Table 1:** Sample Characteristics of the full sample and for the no social anxiety disorder (noSAD group) versus social anxiety disorder group (SAD group).

Metric	Full Sample (noSAD + SAD group combined) *N* = 160)		noSAD group *N* = 96	SAD group *N* = 64
Demographics				
Sex; female *n* (%)		103 (64.38%)	57 (59.37%)	46 (71.78%)
Age *M* (*SD*)		27.76 (9.15)	29.53 (10.39) *	25.11 (6.06) *
Years of Education *M* (*SD*)		15.17 (2.54)	15.02 (3.08)	15.22 (2.62)
Clinical Variables				
*LSAS; M* (*SD*)				
LSAS sum score		40.07 (29.66)	21.45 (18.04) *	67.98 (20.25) *
LSAS anxiety		21.43 (16.74)	10.65 (9.91) *	37.59 (10.77) *
LSAS avoidance		18.64 (13.80)	10.81 (9.35) *	30.39 (10.73) *
LSAS anxiety in interactions		9.09 (7.77)	4.19 (4.50) *	16.44 (5.53) *
LSAS avoidance of interactions		8.99 (6.70)	5.26 (4.64) *	14.59 (5.27) *
LSAS performance anxiety		10.96 (8.44)	5.69 (5.23) *	18.88 (5.70) *
LSAS performance avoidance		9.09 (7.08)	5.38 (5.00) *	14.67 (6.00) *
*Overall psychopathology; M* (*SD*)				
Age of Onset SAD		NA	NA	13.73 (0.76)
BDI-II		10.77 (11.47)	8.35 (11.24) *	14.41 (10.90) *
STAI-t ^a^		46.36 (13.84)	41.90 (14.60) *	51.74 (10.61) *
Fear of negative evaluation (FNEK)		35.65 (6.26)	32.71 (5.70) *	40.06 (3.92) *
Number of psychiatric in-patient treatments		0.30 (0.69)	0.27 (0.66)	0.35 (0.72)
Duration of psychiatric in-patient treatments in months		0.71 (1.91)	0.61 (1.87)	0.87 (1.97)
Number of depressive episodes		1.46 (1.55)	1.23 (1.57) *	1.82 (1.49) *
*Medication; M* (*SD*)				
Medication Load Index		0.38 (0.90)	0.34 (0.82)	0.43 (1.00)

*LSAS* liebowitz social anxiety scale [58], *BDI-II* becks depression inventory [59], *STAI-t* state trait anxiety inventory, trait version [60], *FNEK* fear of negative evaluation scale, German short form [61].

^a^Values are missing for N = 20 participants.

*indicate significant differences between the noSAD and SAD subgroups (p < 0.05).

### MRI data acquisition

MRI data was acquired on a 3-T scanner at the Translational Research Imaging Center in Münster (3 Tesla Hochfeld-MRT MAGNETOM; Prisma, Siemens Medical, Erlangen, Germany). Participants laid supine in the MRI scanner with their head position stabilized. During structural MRI measurements (including T1 and DTI measurements), participants could either observe neutral stimuli of landscapes or keep their eyes closed.

A localizer scan was used to control the head position, which was followed by a high-resolution structural T1-weighted sequence (matrix 256 mm, resolution 1 × 1 × 1 mm³, 192 slices, repetition time [TR] = 2130 ms, echo time [TE] = 2.28 ms, flip angle [FA] = 8 °) with an acquisition time of 4:58 min.

DTI images were acquired (GRAPPA acceleration factor of 2, 56 axial slices, 2.5 mm thickness without a gap, matrix 128 × 128, isotropic voxel size 2.5 × 2.5 × 2.5 mm³, repetition time [TR] = 7300 ms, echo time [TE] = 90 ms) with a duration of 11:47 min. A b-value of 1000 sec/mm² was used for 2 × 30 diffusion-weighed images, with isotropic gradient directions plus five non-diffusion-weighted (b = 0) images.

### Connectome reconstruction

For a detailed description of connectome reconstruction, see Supplementary Material 3. In short, we performed an initial quality check before preprocessing using DTIPrep [36] before correcting for motion and eddy currents using FSL6.0.1 (http://fsl.fmrib.ox.ac.uk/fsl/fslwiki/, FMRIB, Oxford Center for Functional MRI of the Brain, University of Oxford, Department of Clinical Neurology, John Radcliffe Hospital, Oxford, United Kingdom).

The CATO toolbox [37] was used to estimate the structural connectome, using the RESTORE algorithm [38]. This algorithm enhances the accuracy of tensor estimation by identifying and mitigating the effects of outliers and noise artifacts during the tensor fitting process. Fiber reconstruction is then based on deterministic tractography, a rule-based fiber tracking method that follows the dominant diffusion direction to map white matter connections. The 114 nodes were defined using the Cammoun subdivision [39] of the Desikan-Killiany atlas [40]. Thus, for each participant, T1-weighted images were parcellated using Freesurfer and the individual cortical parcellation was then used to define the nodes. To further assess the stability of the effects, we performed the same analyses using Freesurfer’s Desikan-Kiliany atlas [40]. Details can be found in Supplementary Material 4. Edges were defined as white matter (WM) streamlines. Analyses only included edges comprising three or more reconstructed streamlines to balance sensitivity and specificity [41, 42].

### Connectome-based predictive modeling

To test *H1*, connectome-based predictive modeling (CPM) was applied to the full sample of *N* = 160 to identify a structural brain network of trait SA, predicting LSAS sum scores from the participants’ structural connectivity matrices.

Expanding on earlier CPM approaches and as a refinement of our preregistered analysis plan, we used a repeated cross-validation scheme with 10 inner and 100 outer folds (Shen et al., [33]; Winter et al., [43]) and the toolbox NBSPredict [44]. For a detailed description of the toolbox and methodology, see Serin et al. [44]. In short, we applied the toolbox to the participants’ structural connectivity matrices (that is, their structural connectome) to identify key network features predictive of LSAS sum scores through a systematic process of suprathreshold edge selection, model evaluation, and output generation. First, we used suprathreshold edge selection in the outer loop of cross-validation to identify the largest connected components from edges significantly associated with LSAS sum scores (*p* = 0.01; stricter than preregistered), determined using general linear models. Rather than considering isolated edges, NBSPredict applies the Network-Based Statistic (NBS) to identify connected components, defined as sets of suprathreshold edges that are topologically connected within the brain network. This procedure capitalizes on the assumption that disease- or behavior-related effects are more likely to occur in distributed subnetworks than in individual connections while reducing the multiple-comparison burden. The connectivity values of edges in the largest connected component served as predictors for training machine learning models, and their contribution is weighted by the prediction performance in each cross-validation fold.

Hyperparameter optimization (λ₂ regularization) was conducted in an inner loop to maximize prediction performance, including the optimization of the machine learning models, choosing between linear regression and support vector regression. Age and sex were included as covariates of no interest. Model performance was assessed by concatenating the out-of-sample predictions across all outer folds and calculating the Pearson correlation between predicted and observed LSAS scores. Statistical significance of the prediction performance was determined using permutation testing, in which outcome labels were repeatedly shuffled to generate a null distribution against which the observed prediction accuracy was compared. This procedure results in a weighted network which enables visualization of significant edges as a subnetwork, providing interpretable results that preserve the data’s topological structure. This approach ensures robust feature selection while maintaining interpretability and optimizing prediction performance.

We repeated the CPM analyses including only participants that fulfilled criteria for a mental disorder according to the SCID interview, assuming higher levels of SA in a clinical (but not SAD only) subgroup. Consequently, all HC (*N* = 49) were dropped from the analyses, resulting in a sub-sample of *N* = 111. Additionally, we repeated our analysis using the Desikan-Killiany atlas [40].

### Identification of hubs

Follow-up analyses testing *H2* and *H3* were performed in Matlab 2016b. The structural brain networks identified in the previous analyses were analyzed further with a special interest to network graph-theoretical structure (*H2*). Specifically, we identified network hubs using the brain connectivity toolbox [23] and binarized matrices. Hubs in the previously identified networks were defined using local betweenness centrality and nodal degree. In short, a node had to be in the top 15% of the degree distribution as well as the top 15% for betweenness centrality to be considered a hub. For the detailed method, see Supplementary Material 5.

### Global network metrics

The whole-brain structural connectivity matrices were further analyzed using graph metrics [23] to provide insight into the topological organization of the structural connectome in the SAD group versus the noSAD group (*H3*). Measures focused on global network characteristics (global efficiency, clustering coefficient, global connectivity strength and small-worldness). For a comprehensive explanation and summary of these metrics see Rubinov & Sporns [23] and Supplementary Material 6. We then analyzed group differences using ANCOVA with group (SAD vs. noSAD) as the factor and the respective graph metrics as outcome variable. Similarly, we conducted multiple linear regressions with the graph metrics as the outcome variable and LSAS sum scores as the predictor. All analyses were conducted controlling for age and sex. We also repeated these analyses using the Desikan-Killiany atlas [40].

## Results

### Connectome-based predictive modeling

**Fig. 1:**
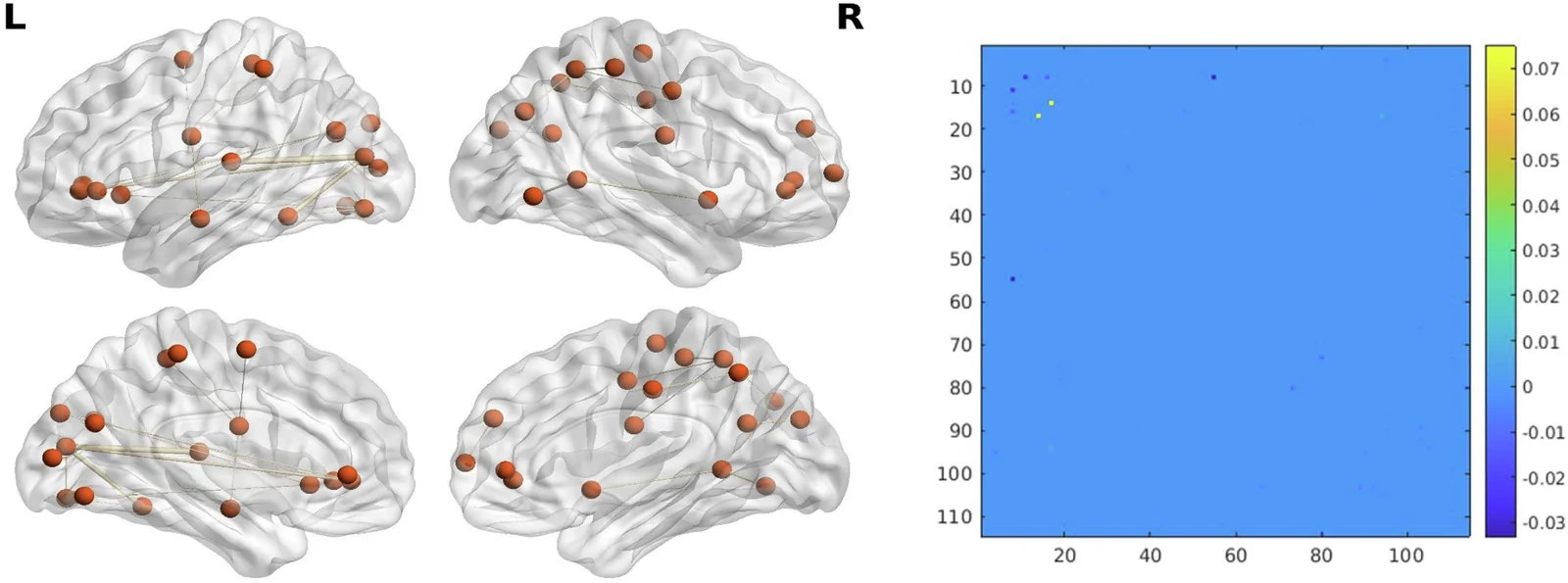
Glass brain and degree-table depiction of edges significantly predicting LSAS sum-scores across the whole sample. The glass brain depiction (left) illustrates the identified brain network spanning across occipital and temporal regions. The degree table (right) indicates the correlation of singular edges with the outcome measure.

CPM revealed a significant network comprising 46 edges that were associated with LSAS sum scores (0.18% of the entire connectome). Most edges were positively associated with LSAS scores (for visual depiction, see Fig. 1.) and predicted LSAS sum-scores with *r* = 0.141 (*CI* = [0.12, 0.2], *p* = 0.003). See Supplementary Material 7 for the detailed report of predictive results.

The identified network comprised a cortical connectivity pattern, with the strongest connections concentrated within visual and temporo-occipital regions. The most prominent positive edge was observed between the left lateral occipital and lingual cortices, indicating a strong association within the visual processing network. In contrast, several negative-weighted connections were identified between fusiform, inferior temporal, and occipital regions, suggesting reduced coupling or opposing connectivity patterns among higher-order visual areas. Overall, the network was characterized by a visual–temporal core with additional weaker connections involving frontal and parietal regions, indicating selective integration between sensory processing regions and distributed cortical areas.

**Fig. 2:**
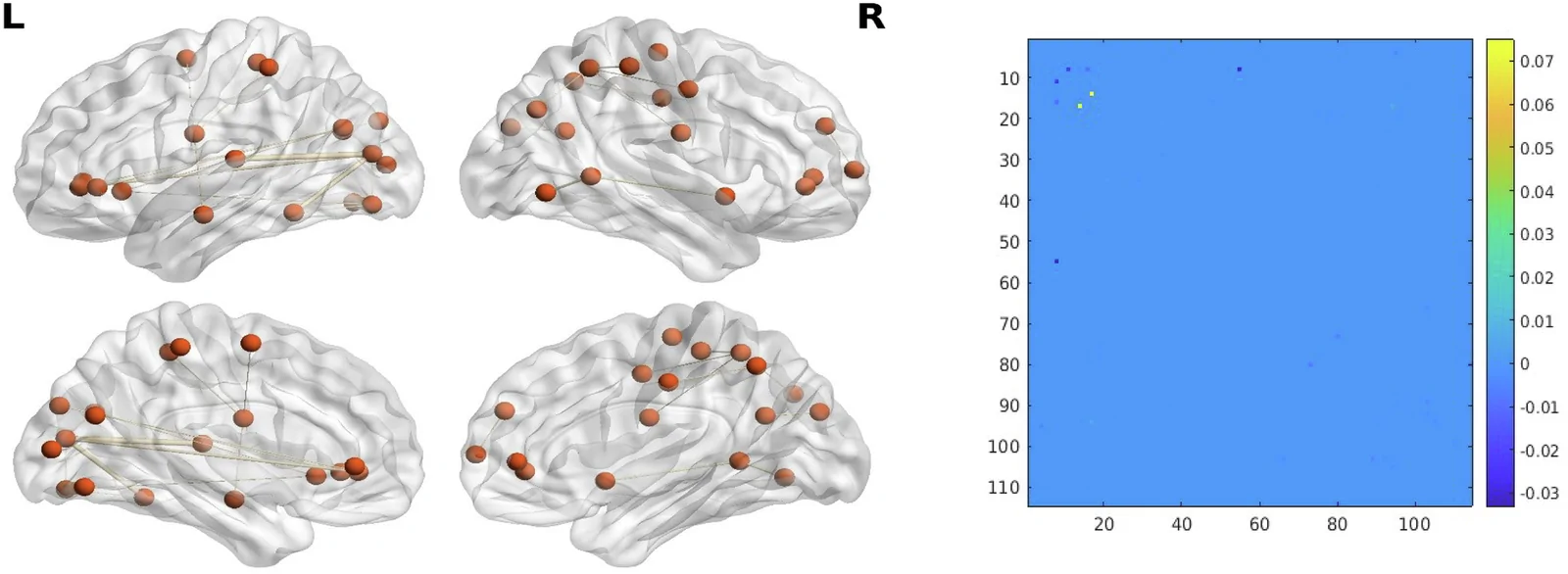
Glass brain depiction of edges significantly predicting LSAS sum-scores in the clinical subset. The glass brain depiction (left) illustrates the identified brain network spanning across occipital and temporal regions. The degree table (right) indicates the correlation of singular edges with the outcome measure.

In the clinical subsample (*N* = 111), we could reproduce the results (see Fig. 2). Using CPM, we identified a brain network comprising 59 edges (0.62% of the entire connectome). Using these selected edges, we were able to predict LSAS sum scores with *r* = 0.172 (*CI* = 0.14, 0.2, *p* < 0.001).

We found similar patterns of connectivity associated with SA using the Desikan-Killiany atlas [40]. For detailed results, see Supplementary Material 8.

### Network hubs

Using both hub definitions combined (top 15% of the degree distribution and for betweenness centrality), no hubs could be identified.

Exploratively, we repeated the analyses for degree only. Using this lenient definition, hubs lied in the left and right precuneus and right superior-parietal cortex for the whole sample. With the more lenient definition, we identified the exact same hubs in the clinical subset.

### Graph-theoretical network characteristics

The ANCOVA revealed that the SAD and noSAD group differed in the number of modules, when correcting for age and sex (*df* = 1(156), *F* = 6.034, *p* = 0.015, partial *η²* = 0.037), but not in any other global metric (see Table 2*)*. Post-hoc tests revealed higher a number of modules in the SAD group compared to the noSAD group.

**Table 2:** Association of clinical severity measures with global graph metrics.

	Clinical severity measure								
	Diagnostic Group (noSAD < SAD)					LSAS sumscore			
	df	*F*	*p*	partial *η²*		df	*F*	*p*	partial *η²*
Number of connections	1 (156)	0.117	0.733	0.001		1[79]	2.474	<0.001*	0.710
Number of streamlines (per connection)		0.007	0.933	<0.001			4.576	<0.001*	0.819
Mean number of streamlines		0.002	0.961	<0.001			1.815	0.004*	0.642
Efficiency		0.200	0.655	0.001			1.694	0.010*	0.626
Normalised efficiency		<0.001	0.985	<0.001			1.311	0.116	0.564
Clustering coefficient		1.512	0.221	0.010			1.461	0.048*	0.591
Normalised clustering coefficient		0.044	0.834	<0.001			1.249	0.163	0.552
Path length		0.061	0.805	<0.001			1.532	0.030*	0.602
Normalised path length		0.149	0.700	0.001			2.015	0.001*	0.665
Small worldness		0.013	0.911	<0.001			1.516	0.033*	0.600
Number of modules		6.034	0.015*	0.037			0.962	0.568	0.487
Modularity		2.538	0.113	0.016			1.216	0.193	0.193

Graph measures were predicted by diagnostic group in an ANCOVA, controlling for age and sex. For the LSAS sum score, multiple linear regressions with age and sex as covariates of no interest were conducted.

Df degrees of freedom, noSAD no social anxiety disorder, SAD social anxiety disorder.

Interestingly, continuous LSAS sum scores were associated with the number of connections, number of streamlines per edge, mean number of streamlines, path length and normalized path length, as well as small worldness, after correcting for age and sex. A comprehensive overview of graph metrics can be found in Table 2.

## Discussion

In this project, we investigated the association of the structural connectome and SA in a transdiagnostic sample to shed light on the complex brain-behavior-relationship causing SA. We were able to predict trait SA in a transdiagnostic sample using CPM and the structural connectome. We added to this finding by identifying hubs and investigating global whole-brain connectivity.

### Connectome-based predictive modeling

In our analyses assessing our first hypothesis (*H1*), we identified a structural brain network associated with SA across the whole sample encompassing both subclinical as well as clinical levels of SA. The identified network was small in size, yet yielded above chance level predictive value. While the effect sizes were small, they were comparable to other effect sizes in the field [45], especially when taking into account that reproducible brain–behavior associations in psychiatry are often small despite large samples and rigorous methodology [46, 47]. These predictive accuracies point towards a relatively stable association between trait SA and the identified structural brain network. The overall stability is supported by the similar network pattern based on the Desikan-Killiany atlas [40] and in our subgroup analyses .

The identified network seems to reflect overall patterns of neural organization associated with individual differences in SA level. In particular, the predictive network was characterized by contributions from occipital and temporal regions involved in visual perception, social cue processing, and higher-order interpretation of sensory information, including lateral occipital, lingual, and fusiform areas. Altered interactions among these regions may reflect differences in the processing or integration of socially relevant visual information, which is a key component of social anxiety [48, 49]. However, given the limited stability of the network, these findings should be interpreted as exploratory and as reflecting potential network-level associations rather than definitive markers of social anxiety. Future studies with larger samples and independent validation are needed to determine the reproducibility and specificity of these connectivity patterns. This network may emerge from developmental processes that shape the architecture of large-scale brain systems involved in socio-emotional functioning. This idea is supported through research in adolescence pointing towards substantial changes in this phase that are also associated with early forms of anxiety (e.g. 50, 51). It suggests that certain connectivity configurations may either predispose individuals to heightened sensitivity in social contexts or result from repeated engagement in socially anxious thought and behavior over time. The emergence of such a network is consistent with the idea that SA is supported by a complex interaction between brain regions, rather than localized abnormalities [18]. These findings raise the possibility that structural brain networks may serve as biomarkers for early identification, risk stratification, or intervention targeting in SA.

In the follow-up analyses only comprising the clinical subgroup, we found a larger brain network comprising both more edges and nodes associated with SA. This network pattern was also reproduced using the Desikan-Killiany atlas [40]. Predictive accuracy was even higher in this clinical subsample. This might be due to the higher homogeneity after the exclusion of HC. While the results were expectedly more pronounced in a subsample with higher to clinically significant trait SA, we could show that these findings generalized to the SA spectrum with our primary analysis. On the other hand, the more pronounced effects in the clinical subsample underline the cumulating effect of higher psychopathology.

### Network hubs

Using our preregistered definition of brain network hubs (top 15% of the degree distribution as well as the top 15% for betweenness centrality), we were not able to identify hubs of the previously identified SA networks (*H2*). This might be due to the small size of the overall network, meaning a lower number of edges and low variance in the number of edges connected to a specific node, and the comparatively small but heterogenous sample. However, explorative follow-up analyses with a more lenient hub definition revealed hubs localized in the left and right precuneus and the right superior-parietal cortex, regions associated with visuospatial cognition and processing, as well as attention and higher cognitive functions [52, 53]. One can speculate that this might be due to the commonly reported hypervigilance for social cues [48, 49], requiring interpretation of spatial positions, movements, and postures of others to assess social dynamics and potential threats. The identified regions are involved in the integration of internally and externally directed attention, visuospatial processing, and higher cognitive functions, including aspects of social information processing and perspective taking. Thus, their involvement could tentatively be related to the heightened monitoring and interpretation of socially relevant cues commonly described in social anxiety. Nevertheless, given that these hubs were only identified under exploratory criteria and considering the limited stability of the network, they should be regarded as candidate regions rather than specific neural markers of social anxiety. Using only a singular measure of brain network centrality to assess potential hubs is not ideal, as centrality is a complex concept best reflected by a combination of different measures. Using degree only might lead to overestimation of the importance of hubs [23]. The identified regions can at best be cautiously regarded as indications of important regions. Additionally, hubs in whole-brain structural networks can be interpreted as nodes, which are interconnected with many other nodes across the network [54], while hubs specific sub-networks associated with traits like SA must be interpreted with care, due to the NBS methodology. NBS uses cluster-level inference, potentially leading to false hub identification (for details on the method, its purpose and pitfalls see [44]).

### Graph-theoretical network characteristics

To gain a deeper understanding of the nature of SA-associated neural alterations, we calculated whole brain graph metrics with specific interest to differences between the noSAD and SAD group on the one hand and associations with SA level across the full sample on the other (*H3*). We found differences between our noSAD and SAD subgroups. Specifically, the number of modules was higher in SAD compared to noSAD sample.

Trait SA was also associated with the number of connections, number of streamlines per edge, mean number of streamlines, path length and normalized path length, as well as small worldness. Number of streamlines per edges, mean number of streamlines and efficiency as well as normalized efficiency were negatively associated with trait SA, while number of connections, path length, normalized path length and small-worldness were positively associated with trait SA.

Taken together, these findings suggest that higher levels of SA may be associated with altered network organization, characterized by increased connectivity alongside reduced efficiency of information transfer. These findings may indicate alterations in the balance between network integration and segregation in individuals with higher levels of SA. Increased path length and small-worldness may suggest a reorganization of network topology, potentially reflecting greater separation between functional modules, whereas the reduced efficiency measures could indicate less effective information transfer across the network. The increase in modular organization observed in the SAD group may further suggest a more segregated network architecture, which could influence the integration of information across distributed systems involved in emotional regulation, attention, and social cognition. However, these interpretations remain tentative, as graph metrics are highly dependent on network construction, and the observed effects may reflect changes within specific SA-associated subnetworks rather than alterations of the entire connectome. One cannot state with certainty if the network effects of SA do or do not generalize across the whole connectome. If they are restricted to an SA-specific subnetwork, one can conclude, that SA-associated alterations in the connectome might not impair the overall connectivity at a global level. When comparing SAD and noSAD group on a whole brain connectivity level, we found results pointing towards categorial differences between those with and without an SAD diagnosis. Those findings are in line with previous research on the structural connectome in anxiety disorders, pointing towards an overall pattern of hyperconnectivity [55].

### Strengths and limitations

This study adds to a line of research by investigating not only SAD-specific characteristics of the structural connectome but also providing insight from a transdiagnostic perspective. The unique sample used is well suited for the investigation of transdiagnostic processes and the in-depth phenotyping provides insight into SA in association with the structural connectome in different populations. To our knowledge, this is the first study investigating the neural foundations of trait SA in a transdiagnostic sample.

Using CPM represents a significant strength of this study, as it enables the identification of brain-behavior relationships in a data-driven, cross-validated framework that enhances the generalizability of our findings. By focusing on predictive performance rather than group level inference, CPM provides a robust approach for linking individual variations in brain structure to social anxiety symptoms.

Yet, sample limitations must be noted. While we were able to include a diverse set of participants with and without mental disorders, our investigation is limited to HC, SAD and MDD. These diagnostic groups had a wide range of SA levels but likely do not fully generalize to the entire population. Specifically, we did not explicitly include a subclinical group. Further, participants in this study reported a wide range of SA levels in self-rating questionnaires but were also characterized by an overall high global functioning (see Table 1). Our findings might not generalize to populations with more severe mental disorders or higher impairments. That we only included limited comorbidities (SAD with MDD) adds to that point. Further, the presence of comorbid depressive symptoms may have contributed to the identified connectivity pattern, raising questions regarding the specificity of the network for social anxiety. At the same time, overlap between anxiety and depressive symptomatology is expected in transdiagnostic samples and is consistent with the dimensional conceptualization of internalizing psychopathology adopted in the present study. Future work should examine the extent to which the identified network reflects shared versus disorder-specific neural mechanisms.

While we were able to enhance generalizability and reproducibility using a data-driven predictive approach, clinical utility was expectedly not improved using our approach. While the observed effects are statistically significant and provide novel insights, their overall magnitude is small. Therefore, these findings should be interpreted with caution and should not be considered established biomarkers or suitable for clinical application at this stage.

Lastly, while larger than most samples investigating neural foundations of SA [18], our sample might still be too small to detect the commonly small effects in psychiatric neuroimaging. An approximative post-hoc sensitivity analysis in G*Power [56] revealed, that our analyses likely would only have been able to detect medium-sized effects (f²=0.15). This simplified approach does not consider power differences across nodes and edges potentially biasing the results and detectability of effects (“differential power”, 57) likely slightly overestimates the effect size that would be detectable as it does not consider evidence that accumulates across multiple connections comprising a subnetwork. Future investigations would also highly profit from a bigger sample size.

### Conclusion

Taking into account these limitations, this study is a first step towards unravelling the neural underpinnings of SA on a transdiagnostic level. Our findings provide evidence for a statistically reliable, albeit modest, association between structural connectivity patterns and social anxiety severity. Using the structural connectome is a useful approach to gain an overview and a new perspective on the complex interplay between brain structure and SA. Future studies should focus on extending our findings by applying a similar approach to an even more diverse sample with greater variability in trait SA across potential subgroups, e.g. a community sample. This could help us better understand the generalizability of SA-associated neurostructural changes, potentially allowing for a refined model of SA(D) and providing a basis for potentially improved treatment by enhancing our understanding of potential target mechanisms.

## Supplementary information

Supplementary Material.
