# Supplementary material for: Identifying a structural brain network for social anxiety: connectome-based predictive modeling and network analyses in a transdiagnostic sample
Source: Transl Psychiatry. 2026 Sep 28;16:495. doi: 10.1038/s41398-026-04466-9 (PMC13619567; doi:10.1038/s41398-026-04466-9)
Supplement: Supplementary file 1 [file 41398_2026_4466_MOESM1_ESM.docx]

***Supplements***

Table of Contents

[**Supplement 1: Study Flow Chart**	2](#_Toc235285472)

[**Supplement 2: Sample Overlap**	2](#_Toc235285473)

[**Supplement 3: Connectome Reconstruction**	3](#_Toc235285474)

[**Supplement 4: Explorative analyses using the Aparc Atlas**	3](#_Toc235285475)

[**Supplement 5: Hub Definition**	3](#_Toc235285476)

[**Supplement 6: Graph Metrics**	4](#_Toc235285477)

[**Supplement 7: Detailed report of Connectome-based predictive Modeling method and results using Lausanne 120**	5](#_Toc235285478)

[**Supplement 8: Detailed report of Connectome-based predictive Modeling results using Aparc**	5](#_Toc235285479)

**Supplement 1: Study Flow Chart**

Figure 1. Flowchart of recruitment efforts and inclusion into the current project. The study design was piloted with n = 15 participants. sMRI = structural MRI, dMRI = diffusion MRI, SAD = social anxiety disorder, SADMDD = co-morbid Major Depression Disorder and Social Anxiety Disorder, MDD = major depression disorder , HC = healthy controls.

**Supplement 2: Sample Overlap**

All participants considered for this project were also part of a project by Slump et al. (in prep, preregistered here: <https://osf.io/gqa5t>), which investigates cortical thickness, surface area, and subcortical brain volumes in social anxiety disorder. N = 28 participants with SAD and N =38 HC (43.8% of participants with SAD and 39.6% of HC in this study) were also used in a recent mega-analysis (Groenewold et al., 2023).

**Supplement 3: Connectome Reconstruction**

An initial quality check was performed prior to preprocessing using DTIPrep (Oguz et al., 2014) with default settings. This step identified artifacts caused by head motion, bed vibration, eddy currents, gradient or slice-wise intensity inconsistencies, and venetian blind artifacts. Participants with artifacts affecting >20% of the images were excluded from the main analyses. For others, only the affected images were removed. Motion and eddy current corrections were conducted using FSL6.0.1 (http://fsl.fmrib.ox.ac.uk/fsl/fslwiki/, FMRIB, University of Oxford).

The structural connectome was estimated using the CATO toolbox (de Lange et al., 2022) with the RESTORE algorithm, which reduces noise artifacts by identifying and removing outliers during tensor estimation. Deterministic tractography reconstructed white matter pathways from diffusion profiles, initiating eight seeds within each voxel. Streamlines were traced voxel-to-voxel along the main diffusion direction, applying stop criteria including fractional anisotropy values below 0.1, sharp turns exceeding 45°, reaching gray matter, or exiting the brain mask. Connectivity matrices were estimated using the Cammoun subdivision (Cammoun et al., 2012) of the Desikan-Killiany atlas (Desikan et al., 2006), which defines 114 nodes. Stability of the findings was further evaluated using Freesurfer’s Aparc atlas (Destrieux et al., 2010; see Supplement X for methods and results). Edges were defined as white matter streamlines, and follow-up analyses included only edges with at least three reconstructed streamlines to balance sensitivity and specificity (de Reus & van den Heuvel, 2013; Zalesky et al., 2016).

**Supplement 4: Explorative analyses using the Aparc Atlas**

The Aparc atlas (Destrieux et al., 2010) is implemented into Freesurfer (Quelle) and is based on an automatic parcellation algorithm. It uses standard anatomical nomenclature to parcellate cortical structures. Based on this, the cortical gyri and sulci are divided into 82 units. Applying this atlas to connectome reconstruction leads to a smaller brain-structural networks consisting of 82 nodes and 3321 potential edges.

**Supplement 5: Hub Definition**

For the estimation of hubs, we used a binarized version of each participant’s connectome (consisting of 0 and 1, 1 meaning an edges is present, 0 an edges is not present). Edges had to be present across 60% of participants to be considered (wie de Reus und van den Heuvel 2013). We then used the Brain Connectivity Toolbox (BCT) for MATLAB (Rubinov & Sporns, 2010) to determine the degree and betweenness centrality for each node to determine hubs. The nodal degree is the number of connections or edges a node has. Edge weights are not considered in the calculation of the degree. Betweenness centrality is defined as the fraction of all shortest paths in the network passing through the node of interest. High scores in betweenness centrality indicate a high relevance of a node within the network. To be considered as a hub, a node had to be in the top 15% of the degree distribution (also used by Liu et al., 2020; Repple et al., 2020; Yan et al., 2018) as well as the 15% for betweenness centrality.

**Supplement 6: Graph Metrics**

For the assessment of hypothesis 3, we calculated a diverse set of graph metrics. An overview of the metrics used can be found below.

| **Metric** | **Definition** | **Interpretation** |
| --- | --- | --- |
| Number of connections (numC) | Total number of connections in a given brain network | Overall connectivity |
| Mean number of streamlines (mean NOS) | Mean number of white matter streamlines in connections present | Overall connectivity |
| Number of Modules | Total number of nonoverlapping groups of nodes, maximizing the number of within-group edges, and minimizing the number of between-group edges. | Functional integration of the connectome |
| Modularity | Degree to which the network is subdivided into modules |  |
| Shortest path length (L) | Average shortest path length between all node pairs | functional integration of the connectome |
| Global efficiency (GE) | Inverse of L | overall communication capacity of the connectome |
| Clustering coefficient (C) | Average probability that a node’s neighbors are also connected | functional segregation of the connectome |
| Normalized shortest path length (L_normalized_) | Normalized^1^ L | Like L, but corrected for differences in numbers of edges between participants |
| Normalized global efficiency (GE_normalized_) | Normalized^1^ GE | Like GE, but corrected for differences in numbers of edges between participants |
| Normalized Clustering coefficient (C_normalized_) | Normalized^1^ C | Like C, but corrected for differences in numbers of edges between participants |
| Normalized number of connections (numC_normalized_) | Normalized^1^ NumC | Like numC, but corrected for differences in number of edges between participants |
| Small-world index (SW) | GE_normalized_/L_normalized_ | Extend to which a network is more clustered than a random network while having a comparable shortest path length |

*Table 1.* The table shows definitions and interpretations of all graph metrics employed in our analyses to assess the topological organization of anatomical brain networks.

^1^To account for differences in the number of edges between participants, we calculated normalized equivalents of L. GE and C. Therefore, 1,000 random networks were generated based on each participant's connectivity matrix. Then, the normalized measures were calculated as the ratio between the measure and the average measure of the random networks.

**Supplement 7: Detailed report of Connectome-based predictive Modeling method and results using Lausanne 120**

We used the NBSPredict toolbox (Serin et al., 2021) to conduct CPM based on the NBS methodology. In our primary analyses, we used the full sample of *N* = 160 participants and their connectome matrices as input. For our analyses, we used a design matrix with LSAS sum-scores as the variable of interest and age and sex as covariates of no interest. We used 10-fold cross-validation and reapeated those steps 100-fold. The p-value threshold was set to 0.01 and the significance of results was assessed using 1000 permutations. Hyper parameters were optimized in an inner loop using 5 steps and grid search. This included the optimization of machine learning models, choosing between linear regression and support vector regression.

For the full sample, the superior model was support vector machine Regression (svmR). Analyses showed a network consisting of 42 nodes and 46 edges, 43 of those edges being positively and 3 being negatively associated with SA. The correlation was r = 0.141 (CI: 0.12, 0.2, permutation: 0.120, p = .003).

Similarly, the superior model was svmR for the clinical sample. Results revealed a correlation of 0.15 (CI: 0.141, 0.168; permutation: 0.120, *p* < 0.001). It consisted of 49 and 59 edges. All associations between nodes and LSAS sumscores were positive.

**Supplement 8: Detailed report of Connectome-based predictive Modeling results using Aparc**

We repeated the CPM analyses for both the full and the clinical subsample. We used the same settings and covariates and yielded comparable results. In both samples, the svmR was the superior model. For the full sample, we yielded as correlation of r = 0.11 (CI: 0.03, 0.14, permutation: 0.120, p = .02). In the clinical sample, the correlation was correlation was r = 0.16 (CI: 0.145, 0.181, permutation: 0.119, p = <.001).
